# Visual Detection of *Cucumber Green Mottle Mosaic Virus* Based on Terminal Deoxynucleotidyl Transferase Coupled with DNAzymes Amplification

**DOI:** 10.3390/s19061298

**Published:** 2019-03-14

**Authors:** Ying Wang, Jing Liu, Hong Zhou

**Affiliations:** Shandong Provincial Key Laboratory of Detection Technology for Tumor Markers, School of Chemistry and Chemical Engineering, School of Life Science, Linyi University, Linyi 276005, China; wangying@lyu.edu.cn (Y.W.); jliu99@126.com (J.L.)

**Keywords:** *cucumber green mottle mosaic virus*, visual detection, DNAzyme, terminal deoxynucleotidyl transferase, cascade amplification

## Abstract

A simple, rapid, and sensitive visual detection method for observing *cucumber green mottle mosaic virus* was reported based on the template-independent polymerization activity of terminal deoxynucleotidyl transferase (TdT), coupled with the cascade amplification of Mg^2+^-dependent DNAzyme and hemin/G-quadruplex DNAzyme. Briefly, the hybridized dsDNA of T1/P1 was cut into two parts at its position of 5′-AA↓CG↑TT-3′ by the restricted enzyme AcII. The longer, newborn fragment originating from P1 was tailed at its 3’-end by oligo dG, and an intact enzymatic sequence of Mg^2+^-dependent DNAzyme was generated. The substrate sequence in the loop segment of the hairpin probe (HP) hybridized with the newborn enzymatic sequence and was cleaved into two parts in the presence of Mg^2+^. The locked G-quadruplex sequence in the stem segment of the HP was released, which catalyzed the oxidation of ABTS^2-^ in the presence of H_2_O_2_, and the resulting solution turned green. A correlation between the absorbance and concentration of T1 was obtained in a range from 0.1 pM to 2 nM, with a detection limit of 0.1 pM. In addition to promoting a lower detection limit and shorter monitoring time, this method also demonstrated an excellent selectivity to single or double nucleotide changes. Therefore, the designed strategy provided a rapid and efficient platform for viral inspection and plant protection.

## 1. Introduction

*Cucumber green mottle mosaic virus* (CGMMV) belongs to the genus *Tobamovirus* (family *Cucurbitaceae*) and has a single-stranded, positive-sense RNA genome of about 6.4 kb [1,2]. The infected cucurbit crops exhibit green mottle, mosaic, and uneven symptoms around growing points. CGMMV is an economically crucial virus which can be transmitted mechanically or through seeds, resulting in a serious reduction in cucumber, melon, watermelon, and similar crops all over the world. The transmission of CGMMV is very fast, due to the frequent international trading of cucurbit seeds [3]. Moreover, CGMMV presents in the leaves, seeds, vines, and roots of infected melon crops and can survive for 10 months in rotting organic matter in the soil. This indicates that it is very difficult to be eliminated from polluted soil. Strict entry detection is one of the effective measures that can be taken to avoid introducing seeds or seedlings of melon crops bearing CGMMV into the soil.

Traditional methods of detection, including serological [4], electron microscope [5] or polymerase chain reaction (PCR) [6] methods, do not satisfy the requirement of a fast, simple, and sensitive process for detecting CGMMV. Such methods require large and expensive instruments and are more laborious, more time-consuming, and less sensitive. Currently, biosensing has become a rising alternative strategy for virus detection, because it is simple, rapid, highly-sensitive, and selective. A variety of methods are employed to detect animal viruses and other diseases including quartz crystal microbalance [7], electrochemiluminescence [8,9], surface plasmon resonance [10,11], loop-mediated isothermal amplification [12,13], fluorescence [14,15], lateral flow [16,17,18], electrochemical techniques [19,20,21], and so on. However, at present, few biosensing methods have been developed for plant virus monitoring.

Visual nucleic acid detection has attracted much attention due to its simplicity, high sensitivity, and visual results [22,23,24,25]. For example, colorimetric detection has been reported for some animal viruses, including H1N1 influenza virus [26,27,28,29], H5N1 influenza virus [30], hepatitis B virus [31,32], Zika virus [33], and Rift Valley Fever Virus [34]. A colorimetric detection method was reported for CGMMV [35] based on unmodified gold nanoparticles, with a limited detection of 30 pg/μL of CGMMV RNA. Though the method is relatively simple and sensitive, it still demonstrates some shortcomings, such as high cost, the instability of AuNPs colloids, and so on. Further development of a simple and rapid strategy, with a higher sensitivity and selectivity, is urgently needed for detecting portable CGMMV.

Terminal deoxynucleotidyl transferase (TdT) catalyzes the random incorporation of dNTPs to the 3′-terminus of DNA molecules and does not need paired DNA as a template, demonstrating a simple, direct, and cost-effective method for single-stranded DNA synthesis [36]. TdT-based detection methods have been developed in many fields, including genome-containing biological targets detection [37], bioluminescent detection of exonuclease I activity [38], highly sensitive fluorometric determination of thrombin [39], uracil-DNA glycosylase detection [40], label-free monitoring of DNA methyltransferase activity [41], aptamer assistant metal ion, protein, and small molecule detection [42], human T-cell lymphotropic virus type II DNA detection [43], microRNA labeling [44], and so on.

DNAzymes are nucleic acids, which can cut specific nucleotide sequences with the help of metal ions as cofactors [45,46,47]. For example, Mg^2+^-dependent DNAzyme, a type of catalytic nucleic acid, has similar features to ribozyme or proteinase. It contains three parts; an enzymatic DNA strand, a substrate DNA strand, and Mg^2+^. In addition to cleaving-DNAzyme, the G-quadruplex-DNAzyme is another type of DNAzyme in which G-rich sequences can fold into a parallel or an antiparallel G-quadruplex in the presence of K^+^ or Pb^2+^. For example, in the presence of hemin, G-quadruplex DNAzyme demonstrates a horseradish peroxidase-mimic activity to catalyze the conversion of a colorless ABTS^2-^ to a green ABTS^·-^, serving as a valuable tool for visual nucleic acid detection [48,49,50].

**Scheme 1:**
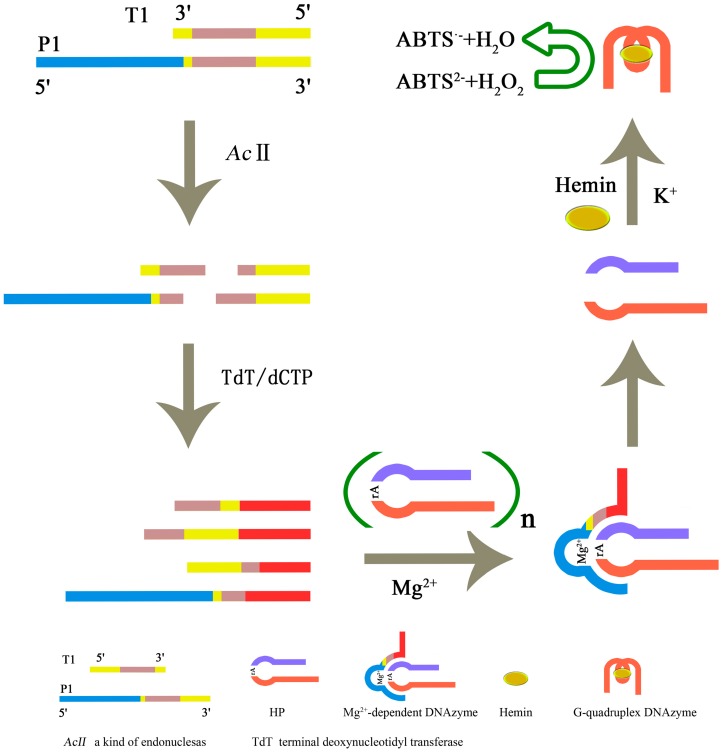
Schematic illustration of terminal deoxynucleotidyl transferase (TdT)-assisted amplification strategy for visual detection of *cucumber green mottle mosaic virus*.

In this study, a novel visual method is proposed, based on TdT activity coupled with Mg^2+^-dependent DNAzyme and hemin/G-quadruplex DNAzyme, for the detection of CGMMV. The oligonucleotide P1 is designed with three regions to position 2375th–2387th in the CGMMV genome including: (1) a partial Mg^2+^-dependent sequence, position 1st–25th (blue in Scheme 1); (2) the recognition sequence of *Ac*II, position 24th–29th (brown in Scheme 1); and (3) a complementary sequence to CGMMV cDNA, position 23rd–35th (brown and yellow in Scheme 1). The hairpin (HP) probe consists of three parts: (1) a sequence of G-quadruplex, position 1st–26th (orange in Scheme 1); (2) a substrate sequence of Mg^2+^-dependent DNAzyme, position 19th–34th; and (3) a stem segment, position 10th–18th and position 35th–43rd. The substrate sequence in HP hybridizes with the enzymatic sequence of Mg^2+^-dependent DNAzyme and is cleaved into two parts in the presence of Mg^2+^. Subsequently, the locked sequence of G-quadruplex in the stem of HP is released. Coupling with the efficient catalysis of hemin/G-quadruplex DNAzyme with ABTS^2-^ as a substrate, the sensitivity of the strategy is improved significantly. The proposed strategy provides a visual detection method for CGMMV, which satisfies both sensitivity and specificity requirements.

## 2. Experimental Section

### 2.1. Material and Reagents

2, 2′-azino-bis (3-ethylbenzothiazoline-6-sulphonic acid) (ABTS) and hemin were obtained from Sigma-Aldrich Co., Ltd. (China). *Ac*II, TdT, and dCTP were obtained from New England Biolabs Ltd. (China). Invitrogen TrackIt Ultra Low Range DNA Ladder was obtained from Thermo Fisher Scientific Ltd. (China). First-strand cDNA Synthesis SuperMix and a plant genome DNA extraction kit were purchased from Tiangen Biotech Co., Ltd. (China). All the other reagents were of analytical grade and were used without further purification. Ultra-pure water with an electrical resistance higher than 18.2 MΩ was obtained by a ULUP-IV system (China) and used throughout the study. All the oligonucleotides were synthesized by Shanghai Sangon Biotechnology Co. Ltd. (China) and are summarized in Table 1.

### 2.2. Instrumentation

Absorbance measurements were performed on a NanoDrop 2000 (Thermo, Waltham, MA, USA). The absorption spectra of the solution were measured in wavelengths ranging from 390 nm to 490 nm. Thermal cycle was performed on a Gene Amp PCR System 9700 Cycler (ABI., Carlsbad, CA, USA). Gel electrophoresis was performed on a BIORAD 1645052 electrophoresis apparatus and a Mini-Protean Tetra Electrophoresis System (Bio-Rad, Berkeley, CA., USA). Gel images were captured by a WD-9413B imaging system (China). The tubes were captured by EOS 200D camera (Canon, Beijing, China).

### 2.3. Procedure for CGMMV Assay

Many CGMMV sequences in NCBI were aligned by DNAMAN (version 7) software. A fragment of nucleotide (nt) 2374th–2387th (CAAACGTTCGGGTT) was conserved in different CGMMV sequences, which has a restriction site of AcII (underlined in Figure S1 in Supplementary data). Both traits of the selected fragment guarantee the sensitivity and specificity of the biosensing assay. Viral cDNA was detected in the biosensing assay as a negative-strand DNA, so we designated a trans-complementary sequence of the fragment as T1 (Figure S1 in Supplementary data).

Hybridization was completed by mixing P1 (1.0 μM) and various concentrations of T1 in the 1×NEB CutSmart buffer for 30 min at a temperature of 37 °C. *Ac*II (5.0 U) was subsequently added to the mixture, and the digestion reaction (10 μL) continued at 37 °C for about 30 min. After digestion, restricted products were added to the buffer solution (50 μL) containing a 1× terminal transferase reaction buffer pack at 0.25 mM CoCl_2_ and 10 U TdT. The mixture remained at 37 °C for about 15 min, followed by inactivation at 85 °C for 3 min. Subsequently, HP and MgCl_2_ (20 mM) were added to the resulting mixture, and the hybridization and cut time of the resulting mixture and HP was about 80 min. Following this, the resolution was incubated with hemin (0.6 µM) and KCl (10 mM) for 15 min at 37 °C to form the hemin/G-quadruplex structure. ABTS (2 mM) and H_2_O_2_ (2 mM) were added to the mixture successively, and the green color of the resulting solution was observed.

### 2.4. Gel Electrophoresis

A 15% polyacrylamide gel electrophoresis analysis of T1/P1 hybrid and its derivatives was done in a constant voltage of 1× TAE at 120 V for about 1 h. Subsequently, the gel was stained in ethidium bromide for 10 min and scanned by the WD-9413B imaging system.

### 2.5. Preparation of Viral cDNA Template from Real Sample

Total plant RNAs (including viral RNAs of CGMMV, *watermelon mosaic virus* (WMV), *melon necrotic spot virus* (MNSV), *soybean mosaic virus* (SMV), *zucchini yellow mosaic virus* (ZYMV), and *squash mosaic virus* (SqMV)) were extracted from different watermelon seedling plants. The concentrations of RNAs were all diluted to 10 pg/μL and stored at −80 °C. Viral cDNA was obtained using First-strand cDNA Synthesis SuperMix with the template of the abovementioned viral RNAs. The typical mixture of the reverse transcription reaction consisted of 10 µL of 2× SuperMix, 2 µL of P3 for CGMMV (or specific primers for WMV, SqMV, SMV, ZYMV, and MNSV), and 8 µL of total plant RNA. The reaction was done at 42 °C for one h and then heated at 85 °C for 3 min to inactivate the reverse transcriptase. Using a plant genome DNA extraction kit, the genomic DNA of watermelon was extracted from healthy watermelon seedlings cv. zaochunhongyu, diluted to 10 ng/μL, and stored at −80 °C.

## 3. Results and Discussion

### 3.1. Principle of CGMMV Assay

The principle of this assay is illustrated in Scheme 1 and involves two steps: (1) hybridization, digestion, and tailing reaction, and (2) activation of the G-quadruplex structure and colorimetric detection. First, T1 was hybridized with P1, and the 5′-AACGTT-3′ sequence in dsDNA was recognized and digested into two parts at the 5′- AA↓CG↑TT-3′ locus by *Ac*II, causing a 5′-CG overhang at the reaction temperature (37 °C). The enzyme-digested products were separated into four ssDNA fragments, as the Tm value of the ssDNA was estimated to be 6 °C–14 °C (Tm = (4 °C) (G/C pairs) + (2 °C) (A/T pairs)). In the presence of TdT and dCTP, the four ssDNA fragments were all tailed by a string of cytidine at their 3′- end, and intact enzymatic sequences of Mg^2+^-dependent DNAzyme were generated. Second, HP and Mg^2+^ were added into the mixture, and the substrate sequence in the loop segment of HP hybridized with the enzymatic sequence to form Mg^2+^-dependent DNAzyme. Subsequently, HP was cleaved into two parts, and the G-quadruplex-forming sequence in the stem segment of HP was released. The recycled enzymatic sequence of Mg^2+^-dependent DNAzyme initiated another round of hybridization/cleaving/separation reactions to release more and more locked G-quadruplex sequences. The G-quadruplex structure was formed by binding hemin in the presence of K^+^, which was able to catalyze the oxidation of ABTS^2-^ by H_2_O_2_. Finally, the solution turned green. However, in the absence of T1, single-strand P1 could not be digested by *Ac*II, and the following intact enzymatic sequence of Mg^2+^-dependent DNAzyme was not synthesized. Moreover, the G-quadruplex sequence remained caged in the presence of H_2_O_2_ and ABTS^2-^, resulting in no color change in the resulting solution.

### 3.2. Feasibility of TdT-Assisted Tailing Reaction

**Figure 1:**
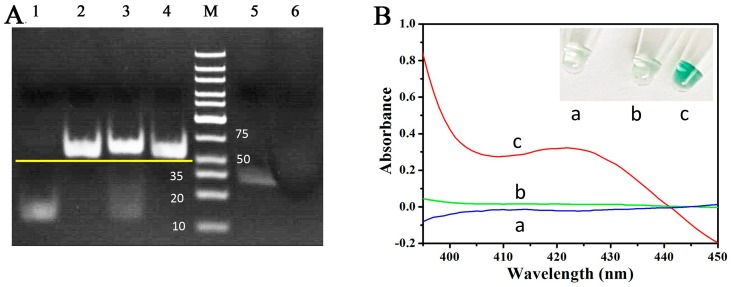
(**A**) Native PAGE analysis of the feasibility of TdT-assisted tailing. Lane 1, T1; lane 2, P1; lane 3, mixture of T1 and P1; lane 4, P1 incubated with 5 U *Ac*II; lane 5, mixture of T1 and P1 incubated with 5 U *Ac*II; and lane 6, mixture of T1 and P1 incubated with 5 U *Ac*II, 100 mM dCTP, and 10 U TdT. (**B**) Colorimetric detection in the solution containing hemin (0.6 µM, 10% DMSO), H_2_O_2_ (2 mM), ABTS (2 mM), and KCl (10 mM), (a) blank control, (b) in the absence and (c) in the presence of T1 (0.2 nM). The insert in B is the image of the color detection with corresponding samples.

In the presence of T1, a partially-hybridized dsDNA was acquired by the base pairing of P1 and T1. The palindromic sequence of 5′-AACGTT-3′ in the dsDNA was recognized and digested into two parts at the 5′- AA↓CG↑TT-3′ loci by *Ac*II at a temperature of 37 °C. As shown in Figure 1A, the distinct bands in lane 1 and lane 2 corresponded to T1 and P1, respectively. When T1 and P1 were all present, a new band was observed in lane 3, showing the presence of the hybridized dsDNA of T1/P1. A new band appeared in line 5, which signifies the completion of the digestion of the T1/P1 hybrid by *Ac*II. However, in the absence of T1, only one band was observed in line 4, indicating that P1, alone, could not be digested by *Ac*II. The underline of lanes 1–4 was used to compare the size of the bands. When TdT and dCTP were added to the *Ac*II-digested mixture, waterfall-like bands were observed in line 6, indicating a successful tailing reaction.

### 3.3. Feasibility of Visual Detection of CGMMV

To demonstrate the feasibility of the colorimetric method for CGMMV detection, both the UV–Vis absorption spectra (395–450 nm) and color changes were analyzed (Figure 1B). No obvious colorimetric signal was observed in (a) the mixture of H_2_O_2_ and ABTS or (b) the sensing system in the absence of T1 at 420 nm. A dramatic increase in the colorimetric signal was observed in (c) the presence of T1 at 420 nm. Only a light green solution was observed in the tube with T1 (c, inserted in the top right corner of Figure 1B), which was in line with that of the UV–Vis absorption monitor. Therefore, these results show that the developed biosensor is able to monitor the concentration of T1.

### 3.4. Optimization of CGMMV Assay

**Figure 2:**
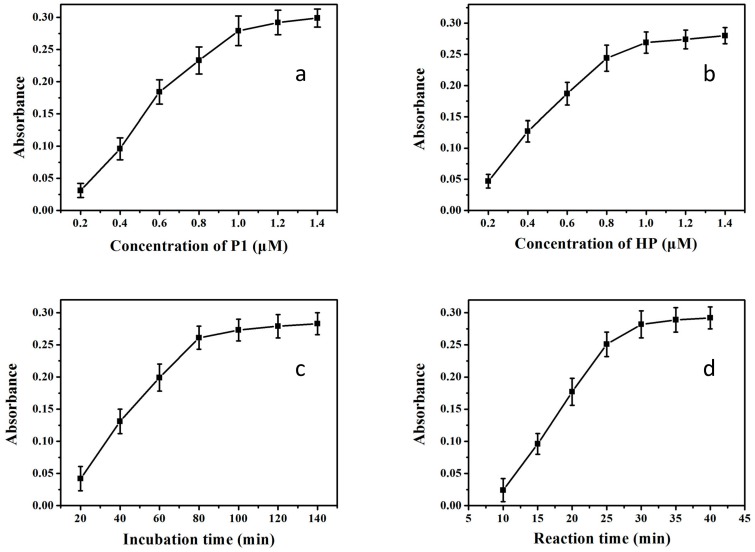
The optimization of concentration of (**a**) P1 and (**b**) HP, (**c**) the incubation time for the resulting mixture and HP, and (**d**) the digestion time for *Ac*II. The concentration of T1 was 0.2 nM. The error bars represent the standard deviations of three repeated experiments and the same below.

The colorimetric signal acquired by peroxidase-mimicking DNAzyme and ABTS depends on several factors including the following: the concentration of P1 and HP, the hybridization time of the resulting mixture and HP, the digestion times of *Ac*II, the reaction time of TdT, and the time required for forming hemin/G-quadruplex DNAzymes. The times required for polymerization by TdT and for forming hemin/G-quadruplex DNAzymes did not exceed 15 min and were scheduled for 15 min in the assay without further optimization. Other experimental conditions affecting the proposed strategy were carefully optimized. For example, 1.0 μM P1, 1.0 μM HP, 80 min of incubation time for the resulting mixture and HP, and 30 min of digestion time for *Ac*II were all chosen as optimal conditions in the following experiments (Figure 2).

### 3.5. Sensitivity of CGMMV Assay

**Figure 3:**
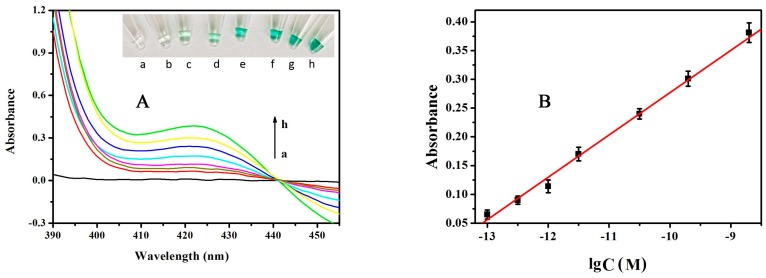
(**A**) Absorbance with various concentrations of T1. The arrow from a to h represents the concentrations of 0 pM, 0.1 pM, 0.25 pM, 1 pM, 2.5 pM, 25 pM, 0.2 nM, and 2 nM. The insert is the image of the color detection of the corresponding concentration of T1. (**B**) The linear correlation between the absorbance and the logarithm of the concentration of T1 (M).

The absorbance of the assay to various concentrations of T1 was verified under optimized experimental conditions. As shown in Figure 3A, an increased concentration of T1 from 0 to 2 nM led to a gradual rise of absorbance at 420 nm. When the concentration of T1 increased, more T1 hybridized with P1 and formed T1/P1 hybrids. The dsDNAs were digested by *Ac*II and tailed by TdT, generating more Mg^2+^-dependent DNAzyme enzymatic sequences, which could hybridize and cleave more HP. More hemin/G-quadruplex DNAzymes were formed, resulting in a significant enhancement of absorbance. The corresponding absorbance was proportional to the logarithm of the concentration of T1 and ranged from 0.1 pM to 2 nM (Figure 3B). The regression equation was A = 1.013 + 0.07356 lgC, with a correlation coefficient of 0.997, in which A and C represented absorbance and concentration of T1, respectively. The detection limit was calculated to be 0.1 pM (about 0.91 μg/mL) by evaluating the average response of the blank control plus three times the standard deviation. Visually, tube b could be recognized by the absence of a light green color reaction, in which T1 was at a concentration of 0.1 pM. The results were consistent with the absorbance at 420 nm (insert on the top right in Figure 3A). The sensitivity of our strategy was also compared with other visually-related detection methods for nucleotide sequences (Table 2), indicating the high performance of our strategy.

**Figure 4:**
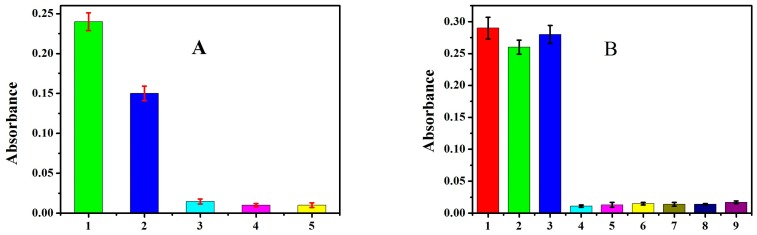
(**A**) Selectivity of the proposed method. (1) T1 (0.2 nM), (2) single-base mismatched target (0.2 nM), (3) two-base mismatched target (0.2 nM), (4) genome of watermelon seedlings (10 ng/μL), and (5) the blank control. (**B**) Selectivity of the assay among six cucurbit crop-related viruses and healthy watermelon seedlings in real sample. (1) T1 (0.2 nM) (2) CGMMV, (3) the mixture of six viral cDNA, (4) *soybean mosaic virus*, (5) *squash mosaic virus*, (6) *zucchini yellow mosaic virus*, (7) *watermelon mosaic virus*, (8) *melon necrotic spot virus*, and (9) genome DNA of healthy watermelon seedlings (10 ng/μL).

Multiple infections are very common in melon crops. The selectivity of the CGMMV assay is therefore critical due to the presence of numerous nucleotide sequences belonging to the host and other viruses. To verify the specificity of the proposed method, T1 (0.2 nM), a single-base mismatched target (SNP-1, 0.2 nM), a two-base mismatched target (SNP-2, 0.2 nM), and the genomic DNA of healthy watermelon seedlings (10 ng/μL) were selected for the following experiments. As shown in Figure 4A, the genomic DNA of healthy watermelon seedlings and SNP-2 had results similar to those of the blank control. In addition, a 61% absorbance signal of SNP-1 was observed, when compared with that of T1, highlighting the great potential of our strategy to be applied in the area of exit–entry detection and plant protection.

The TdT-based assay satisfied detection performance, which is ascribed to the fact that the cascade amplification of the Mg^2+^-dependent DNAzyme and the hemin/G-quadruplex DNAzyme fulfills the strategy with high sensitivity. Moreover, *Ac*II identifies the specific palindromic sequence in P1/T1 hybrids, guaranteeing the assay a high selectivity. In addition, the reaction time of the TdT-based method was no more than 4 h, and the method could be applied in 96-well plates, fulfilling the requirement of high-throughput detection.

The reproducibility of the TdT-based biosensor was investigated via inter-assay and intra-assay methods. The intra-assay reproducibility was completed with 0.2 nM T1 for every 5 h period within the same day, and the relative standard deviations (RSD) value was calculated at 4.2%. Similarly, the inter-assay reproducibility was measured with 0.2 nM T1 on three consecutive days, and an RSD value of 3.8% was obtained, showing an acceptable reproducibility outcome for the proposed method. The stability of the TdT-based biosensor was also measured using 0.2 nM T1, for a parallel determination of 5 times, and the RSD value was calculated at 4.40%. The results show that the strategy has favorable stability.

### 3.6. Challenged with Plant Samples

Total plant RNA (including viral RNA) and the genomic DNA of watermelon were all extracted from watermelon seedlings. All six cucurbit crop-related total plant RNAs were 10 pg/μL, including CGMMV, WMV, MNSV, SMV, ZYMV, and SqMV, respectively. Viral cDNAs were acquired by reverse transcription. As shown in Figure 4B, T1 (0.2 nM), CGMMV cDNA and the mixture of six viral cDNA showed a high absorbance signal, while no obvious signals were found in the healthy seedlings of the watermelon, MNSV, SMV, WMV, ZYMV, and SqMV samples. The results confirmed that the assay was competent in detecting CGMMV within a complex mixture, showing the potential for this method to be applied in the areas of plant inspection for imports and exports and for plant protection.

## 4. Conclusions

In this study, a simple, rapid, sensitive, and specific visual method for the detection of *cucumber green mottle mosaic virus*, based on TdT, Mg^2+^-dependent DNAzyme and hemin/G-quadruplex DNAzyme, was established. Employing the template-independent polymerization function of TdT, an intact enzymatic sequence of Mg^2+^-dependent DNAzyme was generated. Coupled with hemin/G-quadruplex DNAzyme, the cascade amplification strategy demonstrated significant sensitivity with a detection limit of 0.1 pM. The restricted recognition site of *Ac*II in P1 guaranteed the excellent selectivity of the assay from a single nucleotide change and other cucurbit crop-related viruses. Thus, the cascade amplification strategy avoided the intricate operations, cumbersome instruments, and sophisticated designs previously required for detection, providing the potential for agricultural inspection applications and customs quarantine.

## Figures and Tables

**Table 1:** Sequence of oligonucleotides designed in the present study.

Note	Sequence (5′-3′)
P1	ACACACAGCGATCACCCATGTTAAACGTTCGGGTT
T1	AACCCGAACGTTTG
SNP-1	AACCCGAACGTTAG
SNP-2	AACCCGAACGTAAG
Hairpin (HP)	TTTTGGGTTGGGCGGGATGGGTTTATrAGGTGTGTATCCCGCCC
P3	ATGCGAGTGGTATCGTCACT
Oligo-dT	TTTTTTTTTTTTTTTTTTTTTTTT

**Table 2:** Comparison of visually related sensors for nucleotide sequence detection.

Analytical Method of Nucleotide Detection	Detection Limit	Reference
Detection of unamplified pathogen DNA by dextrin-capped gold nanoparticles	2.94 fM	[51]
HBV gene detection by silver-coated glass slide and DNAzyme	0.2 nM	[49]
*Pseudomonas aeruginosa* ETA gene detection by gold nanoparticles DNA probe and endonuclease enzyme	9.899 ng/mL	[52]
Detection of breast cancer 1 by a 3D DNA nanostructured reporter probe	10 fM	[53]
*Salmonella Enteritidis* detection by DNA aptamer	10^3^ CFU/mL	[54]
Gold nanoparticles-based method for CGMMV detection	30 pg/μL	[35]
Exonuclease III-based colorimetric DNA detection	1 pM	[55]
Detection of CGMMV by TdT coupled with DNAzymes amplification	0.1 pM (0.91 μg/mL)	Our strategy

## Supplementary Materials

The following are available online at https://www.mdpi.com/1424-8220/19/6/1298/s1, Figure S1: Alignment of ten CGGMV sequences in the region of 2330 to 2400 nt. Fragment of nt 2374th–2387th was conserved in these CGMMV sequences, and recognition site of AcII was underlined in trans-complementary sequence of T1.
