# Supplementary material for: Visual Detection of Cucumber Green Mottle Mosaic Virus Based on Terminal Deoxynucleotidyl Transferase Coupled with DNAzymes Amplification
Source: Sensors (Basel). 2019 Mar 14;19(6):1298. doi: 10.3390/s19061298 (PMC6471243; doi:10.3390/s19061298)
Supplement: Supplementary file 1 [file sensors-19-01298-s001.pdf]

sensors

Communication

Visual Detection of *Cucumber Green Mottle Mosaic Virus* Based on Terminal Deoxynucleotidyl Transferase Coupled with DNAzymes Amplification

Ying Wang, Jing Liu and Hong Zhou *

Shandong Provincial Key Laboratory of Detection Technology for Tumor Markers, School of Chemistry and Chemical Engineering, School of Life Science, Linyi University, Linyi 276005, China; wangying@lyu.edu.cn (Y.W.); jliu99@126.com (J.L.)

* Correspondence: zhouhong@lyu.edu.cn; Tel.: +86-539-7258627

Received: 24 January 2019; Accepted: 12 March 2019; Published: date

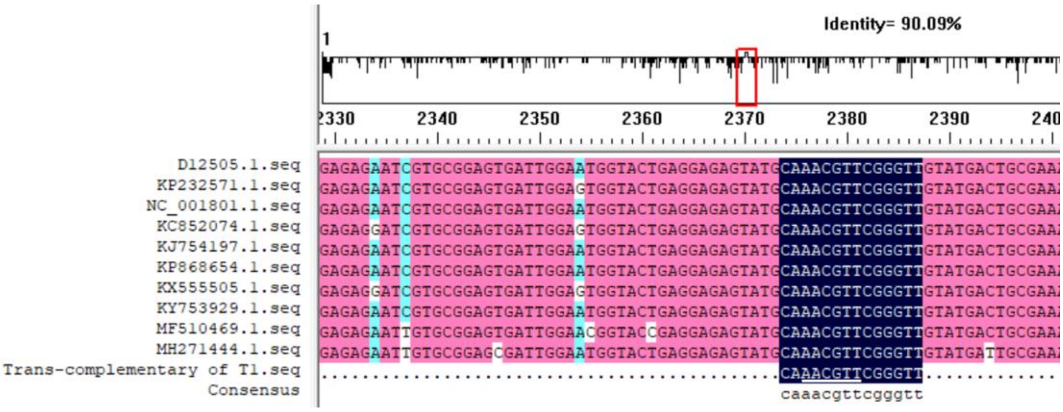

Figure S1. Alignment of ten CGGMV sequences in the region of 2330 to 2400 nt. Fragment of nt 2374th–2387th was conserved in these CGMMV sequences, and recognition site of AcII was underlined in trans-complementary sequence of T1.
